# Clinical Implications of Nutritional Intake in Patients With Esophageal Squamous Cell Carcinoma Receiving Chemoradiotherapy and Neoadjuvant Chemotherapy

**DOI:** 10.1002/cam4.71714

**Published:** 2026-03-12

**Authors:** Yumiko Tamai, Atsushi Yamada, Chikatoshi Katada, Masashi Tamaoki, Motoo Nomura, Akira Yokoyama, Hiroyuki Inoo, Kota Fujii, Katsuyuki Sakanaka, Shigeru Tsunoda, Shinya Ohashi, Yoshitaka Nishikawa, Mayumi Inoue, Kazutaka Obama, Takashi Mizowaki, Manabu Muto

**Affiliations:** ^1^ Department of Medical Oncology, Graduate School of Medicine Kyoto University Kyoto Japan; ^2^ Department of Radiation Oncology and Image‐Applied Therapy, Graduate School of Medicine Kyoto University Kyoto Japan; ^3^ Department of Surgery, Graduate School of Medicine Kyoto University Kyoto Japan; ^4^ Preemptive Medicine and Lifestyle Related Disease Research Center, Kyoto University Hospital Kyoto Japan; ^5^ Department of Health Informatics, Kyoto University School of Public Health Kyoto Japan

**Keywords:** body mass index, esophageal squamous cell carcinoma, nutritional intake, psoas muscle mass index, serum albumin level

## Abstract

**Background:**

Nutritional management plays a pivotal role in the treatment of esophageal squamous cell carcinoma (ESCC). However, the impact of nutritional intake on patients‘ nutritional status during treatment remains unclear.

**Methods:**

This retrospective study aimed to compare the nutritional status of patients with ESCC with that of healthy controls and evaluate how nutritional intake affects patients undergoing chemoradiotherapy (CRT) and neoadjuvant chemotherapy (NAC). Clinical data from 166 patients with ESCC who received two courses of chemotherapy with 5‐fluorouracil plus cisplatin (109 in the CRT group and 57 patients in the NAC group) were examined, along with 166 matched healthy controls.

**Results:**

Compared with healthy controls, patients with ESCC had poorer nutritional status as assessed using serum albumin levels, body mass index (BMI), and psoas muscle mass index (PMI), which declined further during treatment. Notably, patients who achieved sufficient energy and protein intake maintained better nutritional status. Although no significant differences were observed in nutritional intake between the groups, the CRT group was associated with greater decreases in serum albumin levels, BMI, and PMI than the NAC group.

**Conclusion:**

Adequate nutrition intake may mitigate the deterioration of the nutritional status in patients with ESCC during treatment. Moreover, nutritional requirements may differ depending on the treatment modality, such as CRT and NAC. These findings underscore the importance of individualized nutritional management in the care of patients with ESCC.

## Graphical abstract

In this retrospective study, we demonstrated that adequate nutritional intake attenuated the decline in nutritional status among patients with esophageal squamous cell carcinoma (ESCC) receiving chemoradiotherapy and neoadjuvant chemotherapy. Moreover, we found that nutritional requirements may depend on the treatment modality. These findings highlight the importance of individualized nutritional support, which may improve the outcomes of patients with ESCC.

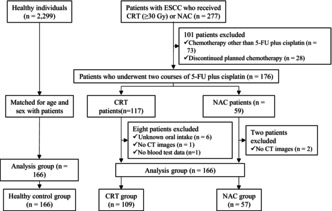

**Abbreviations**
5‐FU: 5‐Fluorouracil
BMI: Body mass index
CRT: Chemoradiotherapy
ESCC: Esophageal squamous cell carcinoma
ESPEN: European Society for Clinical Nutrition and Metabolism
NAC: Neoadjuvant chemotherapy
PMI: Psoas muscle mass index

## 1 Introduction

Recent evidence highlighted the critical role of nutritional status in treatment tolerance, functional capacity, and survival outcomes among patients with cancer. Malnutrition—manifested as cancer‐associated weight loss [1], muscle depletion (sarcopenia) [2, 3], and hypoalbuminemia [4]—has been associated with poor prognosis and increased treatment‐related toxicity across various cancer types. Gastrointestinal cancers, particularly esophageal cancer, carry a high risk of nutritional impairment due to tumor‐related dysphagia and treatment‐induced adverse effects, such as mucositis, nausea, and anorexia.

To optimize nutritional care for patients with cancer, the European Society for Clinical Nutrition and Metabolism (ESPEN) recommends early nutritional risk screening and individualized interventions, including oral nutritional supplements, symptom management, and nutritional counseling [5]. These strategies have been shown to help preserve body weight, maintain skeletal muscle mass, and improve the quality of life in patients with various cancer types, including those with gastrointestinal cancers [6, 7, 8, 9].

Esophageal squamous cell carcinoma (ESCC), which is the predominant histological subtype in East Asian countries, including Japan [10], is often associated with significant pretreatment malnutrition. Treatment modalities, such as definitive chemoradiotherapy (CRT), neoadjuvant CRT, and neoadjuvant chemotherapy (NAC), may further exacerbate nutritional decline through mucosal injury, nausea, and anorexia [11, 12, 13]. Although baseline malnutrition markers, such as serum albumin levels, body mass index (BMI), and skeletal muscle mass, have been linked to the prognosis of patients with ESCC [14, 15, 16, 17], the effects of energy and protein intake during treatment on these parameters remain unclear. Moreover, limited information is available regarding the nutritional status changes during CRT and NAC or whether these changes differ by treatment modality.

In this study, we retrospectively compared the nutritional status of patients with ESCC who underwent CRT or NAC with that of matched healthy controls. We also investigated how energy and protein intake during treatment affected changes in serum albumin levels, BMI, and psoas muscle mass index (PMI). We aimed to obtain insights to aid the development of individualized nutritional strategies in the clinical management of ESCC.

## 2 Methods

### 2.1 Study Subjects

**FIGURE 1:**
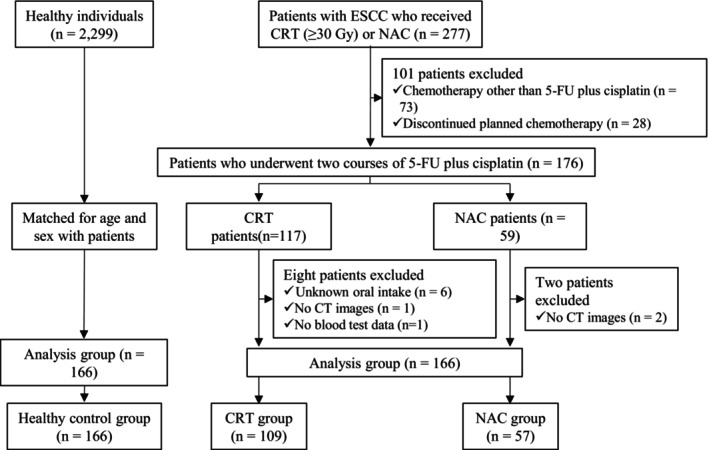
Study flowchart. 5‐FU, 5‐fluorouracil; CRT, chemoradiotherapy; ESCC, esophageal squamous cell carcinoma; NAC, neoadjuvant chemotherapy.

We retrospectively analyzed 166 consecutive patients with ESCC who underwent CRT or NAC at the Kyoto University Hospital between September 2013 and December 2018. The CRT group (*n* = 109) received chemotherapy consisting of 5‐fluorouracil (5‐FU) and cisplatin in combination with radiation therapy at a total dose of at least 30 Gy. Of these, 65 patients received 700 mg/m^2^ 5‐FU on days 1–4 and 70 mg/m^2^ cisplatin on day 1, whereas 20 received 1000 mg/m^2^ 5‐FU on days 1–4 and 75 mg/m^2^ cisplatin on day 1. These regimens were administered twice every 4 weeks. An additional 24 patients in the CRT group received various doses of 5‐FU and cisplatin, primarily reduced cisplatin doses due to renal dysfunction. Among the patients in the NAC group (*n* = 57), 48 received 5‐FU (800 mg/m^2^, days 1–5) and cisplatin (80 mg/m^2^, day 1) twice every 3 weeks, while nine patients received reduced doses of 5‐FU and/or cisplatin. Patients who received chemotherapy regimens other than 5‐FU plus cisplatin (*n* = 73), discontinued the planned chemotherapy (*n* = 25), had undocumented oral intake (*n* = 6), had no blood test data (*n* = 1), or lacked computed tomography images (*n* = 3) were excluded. A total of 166 age‐ and sex‐matched healthy controls who had no signs or findings suggestive of any malignant tumors were selected from individuals who underwent medical checkups at the Preemptive Medicine and Lifestyle Related Disease Research Center at Kyoto University Hospital between June 2016 and December 2018. When an exact age match was not available, a 1‐year age difference was permitted. Among the eligible healthy individuals who met these criteria, those with the closest hospital identification numbers to the corresponding cases were selected as controls (Figure 1). This study was approved by the Ethics Committee of Kyoto University Graduate School and Faculty of Medicine (R3530‐1). Written informed consent was waived by the Ethics Committee because the study used pseudonymized data and provided participants with the opportunity to opt out.

### 2.2 Collection of Clinical Data and Nutrition‐Related Variables

Clinical data were retrospectively collected from the electronic medical records. Cancer staging was assessed according to the Union for International Cancer Control TNM Classification of Malignant Tumors (8th edition) [18]. BMI was calculated using body weight and height at the start of treatment and after the completion of the two treatment courses. PMI was calculated both before and after treatment by dividing the cross‐sectional area of the psoas muscle by the square of the patient's height (m^2^). The psoas muscle area was measured at the level of the third lumbar vertebra using computed tomography images analyzed with the three‐dimensional image analysis software Synapse Vincent Ver 5.5 (Fujifilm, Tokyo, Japan). The cutoff values of PMI were set at 6.36 cm^2^/m^2^ for men and 3.92 cm^2^/m^2^ for women based on a previous study in healthy Japanese individuals [19]. Changes in serum albumin levels, BMI, and PMI before and after CRT and NAC were expressed as a “post/pre ratio,” calculated by dividing the posttreatment values by the corresponding pretreatment values. Data on energy (kcal) and protein (g) intake, including oral intake and parenteral and enteral nutrition, were obtained from electronic medical records [20] and averaged over 6 days during the second course of chemotherapy, when patients were more likely to experience treatment‐related reductions in nutritional intake. According to the ESPEN guidelines for patients with cancer [5], energy and protein intake of < 25 kcal/kg/day and < 1 g/kg/day was defined as insufficient, whereas intake of ≥ 25 kcal/kg/day and ≥ 1 g/kg/day was considered sufficient, respectively.

### 2.3 Statistical Analysis

Differences in categorical variables between patients with ESCC and healthy controls, and between the CRT and NAC groups, were analyzed using Fisher's exact test. Continuous variables are expressed as medians with ranges for age or as medians with interquartile range for energy and protein intake and compared using the Mann–Whitney *U* test. The Wilcoxon signed‐rank test was used to assess changes in the nutritional parameters before and after treatment. Spearman's rank correlation was used to assess the relationship between energy and protein intake and changes in nutritional parameters. All *p*‐values were two‐sided, and a *p* < 0.05 was considered statistically significant. The Bonferroni correction was used for multiple comparisons. All statistical analyses were performed using GraphPad Prism 5 software (Boston, MA, USA).

## 3 Results

### 3.1 Characteristics and Nutritional Status of Patients With ESCC

**FIGURE 2:**
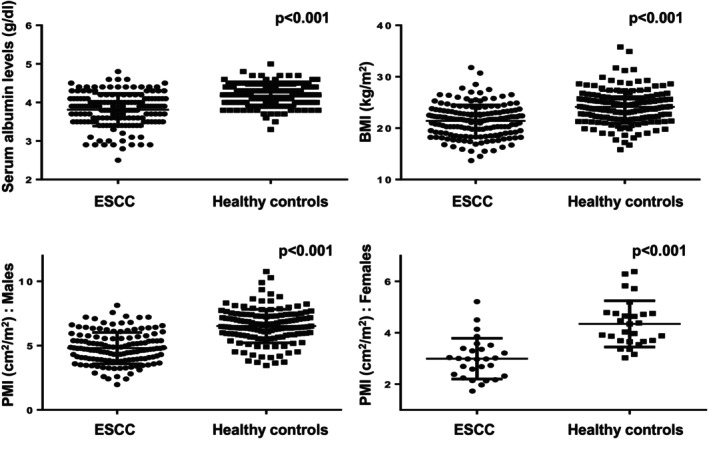
Comparison of serum albumin levels, BMI, and PMI between patients with ESCC and healthy controls. Statistical analyses were performed using the Mann–Whitney *U* test. BMI, body mass index; ESCC, esophageal squamous cell carcinoma; PMI, psoas muscle mass index.

The baseline characteristics of patients with ESCC and age‐ and sex‐matched healthy controls are summarized in Table 1. The median age of the 166 patients with ESCC was 66 years, and 83.1% were male. A larger number of healthy controls had dyslipidemia than patients with ESCC, whereas a history of smoking and alcohol consumption was more common among patients with ESCC. The baseline characteristics between the CRT and NAC groups were generally well balanced among patients with ESCC, except for a significantly higher proportion of patients with stages I and IV disease in the CRT group than in the NAC group. Patients with ESCC had significantly lower serum albumin levels, BMI, and PMI than healthy controls (Figure 2).

**TABLE 1:** Characteristics of patients with ESCC and age‐and sex‐matched healthy controls.

	ESCC		*p* ^a^	Healthy controls	*p* ^b^
	NAC (*n* = 57)	CRT (*n* = 109)		(*n* = 166)	
Age (median range)	66 (40–78)	67 (45–79)	0.201	66 (40–79)	0.990
Sex					
Male/Female	49/8	89/20	0.522	138/28	1
Hypertension					
Yes/No	27/30	38/71	0.133	71/95	0.577
Dyslipidemia					
Yes/No	3/54	17/92	0.077	48/118	< 0.001
Diabetes					
Yes/No	7/50	13/96	1	38/128	0.014
Smoking history					
Never/Ever/Unknown	8/49/0	15/93/1	1	58/108/0	< 0.001
Alcohol drinking history					
Never/Ever/Unknown	4/49/4	3/100/6	0.354	35/131/0	< 0.001
Stage					
I/II/III/IVa/IVb	0/33/20/1/3	26/8/15/18/42	< 0.001		

Abbreviations: CRT, chemoradiotherapy; ESCC, esophageal squamous cell carcinoma; NAC, neoadjuvant chemotherapy.

^a^ Comparison between the NAC and CRT groups.

^b^ Comparison between patients with ESCC and healthy controls. Differences in categorized variables were analyzed using Fisher's exact test, whereas continuous variables were compared using the Mann–Whitney *U* test. Cancer staging was assessed clinically based on the Union for International Cancer Control TNM Classification of Malignant Tumors (8th edition).

### 3.2 Changes in Nutritional Status After Treatment

**FIGURE 3:**
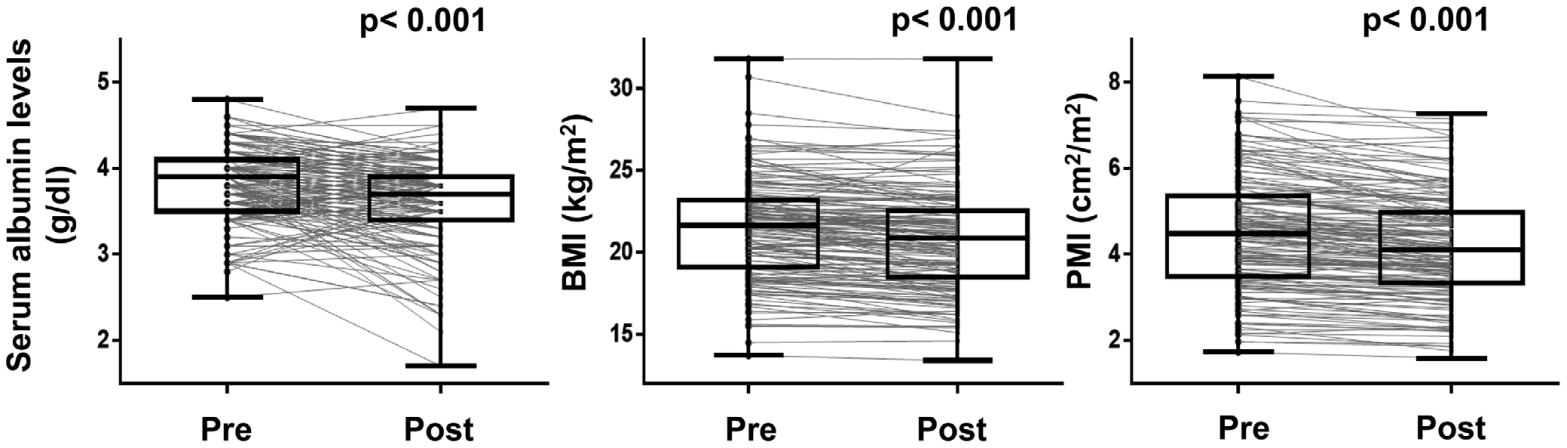
Changes in nutritional parameters during treatment in patients with ESCC. Serum albumin levels, BMI, and PMI were compared between the day of the start of treatment (pre) and after the end of treatment (post). Statistical analyses were performed using the Wilcoxon signed‐rank test. BMI, body mass index; CRT, chemoradiotherapy; ESCC, esophageal squamous cell carcinoma; NAC, neoadjuvant chemotherapy; PMI, psoas muscle mass index.

We next evaluated changes in the nutritional status of patients with ESCC after treatment. Serum albumin levels, BMI, and PMI decreased significantly after treatment (Figure 3). These reductions were observed in both CRT and NAC groups when analyzed separately (Figure S1).

### 3.3 Association Between Nutritional Intake During Treatment and Changes in Nutritional Status

**FIGURE 4:**
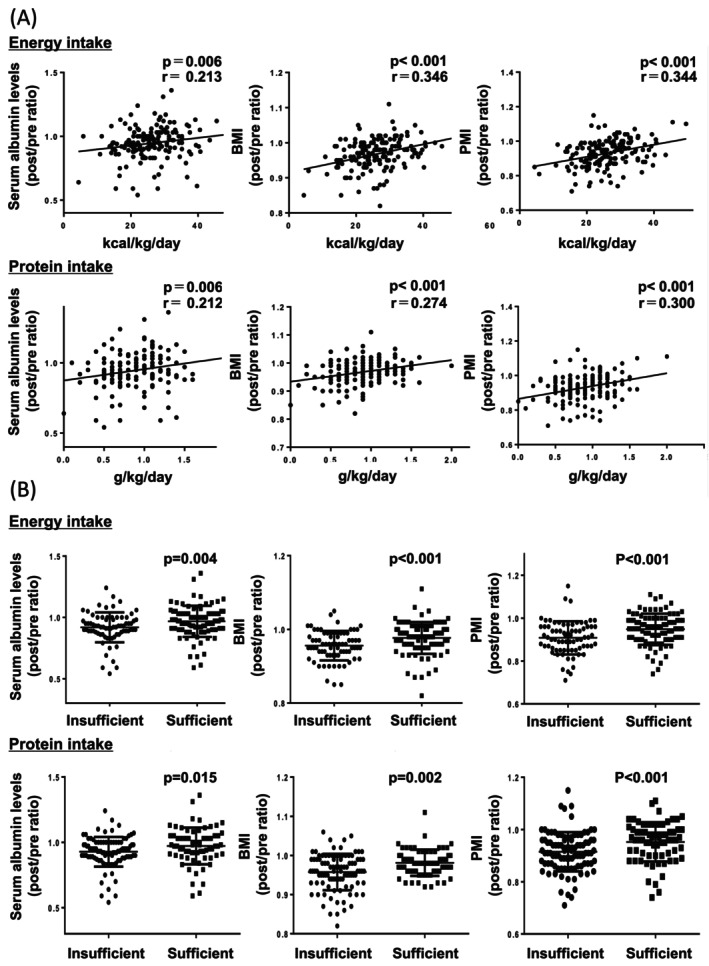
Impact of nutritional intake on treatment‐related changes in nutritional status of patients with ESCC. (A) Correlations between energy and protein intake per kg of body weight during treatment and post/pre ratio of serum albumin levels, BMI, and PMI of patients with ESCC were assessed using Spearman's rank correlation coefficient (rho). (B) Comparison of the post/pre ratio of serum albumin levels, BMI, and PMI between patients with ESCC with insufficient and sufficient energy and protein intake. According to the ESPEN guidelines, patients with energy intake of < 25 kcal/kg/day and protein intake of < 1 g/kg/day were grouped into “insufficient,” whereas those with energy intake of ≥ 25 kcal/kg/day and protein intake of ≥ 1 g/kg/day were considered “sufficient.” Statistical analyses were performed using the Mann–Whitney *U* test. BMI, body mass index; ESCC, esophageal squamous cell carcinoma; ESPEN, European Society for Clinical Nutrition and Metabolism; PMI, psoas muscle mass index.

Energy and protein intake during treatment showed a weak but positive correlation with the post/pre ratio of serum albumin levels, BMI, and PMI in patients with ESCC (Figure 4A). Similar trends were observed in both CRT and NAC groups when analyzed separately (Figure S2). We further examined whether adequate nutritional intake affected changes in nutritional status. Patients who met the ESPEN‐recommended targets for energy and protein intake had higher post/pre ratios of serum albumin levels, BMI, and PMI than those with insufficient intake (Figure 4B). These trends remained consistent when the CRT and NAC groups were analyzed separately (Figure S3).

### 3.4 Comparison Between the CRT and NAC Groups

**FIGURE 5:**
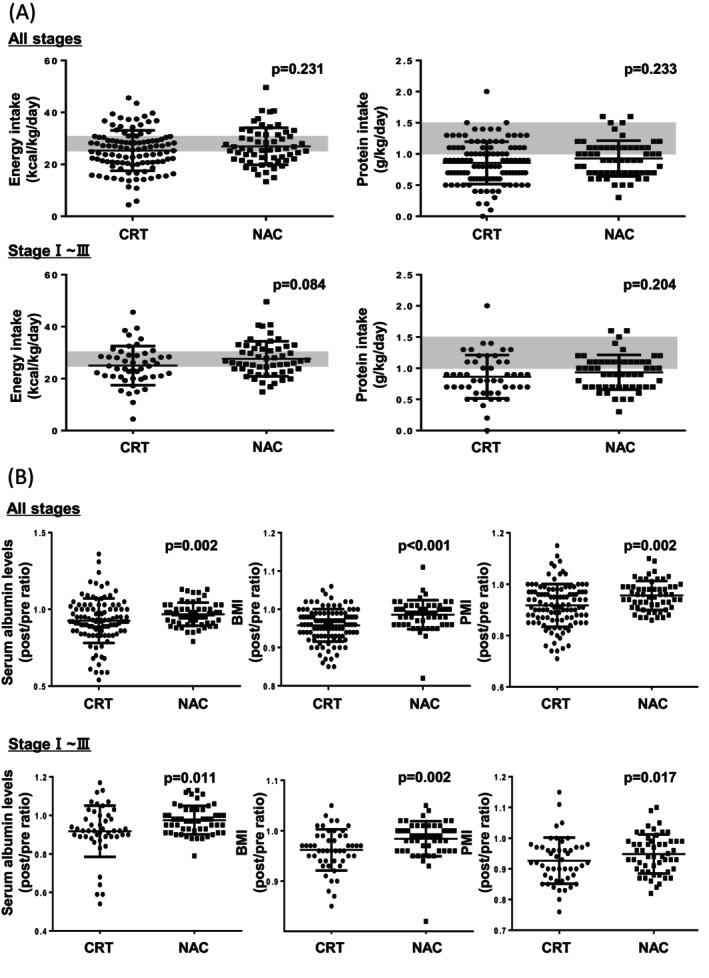
Comparison of nutritional intake and treatment‐related changes in nutritional status between the CRT and NAC groups of patients with ESCC at all stages and those with stage I to III disease. (A) Energy and protein intake were compared between the CRT and NAC groups using the Mann–Whitney *U* test. The gray horizontal bars indicate the range of energy and protein intake recommended by the ESPEN guidelines. (B) Post/pre ratios of serum albumin levels, BMI, and PMI were compared between the CRT and NAC groups. Post/pre ratios were calculated as serum albumin levels, BMI, or PMI after the end of treatment divided by the respective values on the day of treatment initiation. Statistical analyses were performed using the Mann–Whitney *U* test. BMI, body mass index; CRT, chemoradiotherapy; ESCC, esophageal squamous cell carcinoma; ESPEN, European Society for Clinical Nutrition and Metabolism; NAC, neoadjuvant chemotherapy; PMI, psoas muscle mass index.

The median energy and protein intake per kg of body weight during treatment were 26.1 (20.7–30.4) kcal/kg and 0.9 (0.6–1.1) g/kg in the CRT group and 26.0 (22.5–31.4) kcal/kg and 0.9 (0.7–1.1) g/kg in the NAC group, respectively, with no significant differences between the groups. According to the ESPEN criteria, 47% and 39% of patients in the CRT and NAC groups, respectively, had insufficient energy intake, whereas 61% and 53%, respectively, had insufficient protein intake. Although the CRT group included more patients with stage IV disease, which can potentially confound nutritional intake, no significant differences in energy and protein intake were observed between the groups when the analysis was restricted to patients with stage I–III disease (Figure 5A). Finally, we compared the changes in nutritional status between the CRT and NAC groups. The CRT group exhibited greater reductions in serum albumin levels, BMI, and PMI than the NAC group, and these differences remained evident even when restricting the analysis to patients with stage I–III ESCC (Figure 5B).

## 4 Discussion

The present study found that sufficient energy and protein intake, as recommended by the ESPEN guidelines, helped mitigate the deterioration of nutritional status in patients with ESCC undergoing CRT and NAC. Patients with ESCC had lower serum albumin levels, BMI, and PMI than healthy controls before the initiation of treatment, and the levels of these nutritional parameters declined further during treatment. Given that impaired nutritional status has been associated with poor prognosis in patients with ESCC [15, 16, 17, 18, 19, 21, 22], strategies to maintain nutritional status may be essential in the clinical management.

In our analysis, both energy and protein intake showed weak but positive correlation with changes in serum albumin levels, BMI, and PMI during treatment. Approximately 40% of patients had insufficient energy intake, and over half had insufficient protein intake during CRT and NAC according to the ESPEN criteria. These findings highlight the need for proactive nutritional interventions to ensure adequate energy and protein intake during CRT and NAC. Importantly, patients who met the ESPEN‐recommended thresholds demonstrated better maintenance of nutritional parameters than those who did not.

Interestingly, the decreases in serum albumin levels, BMI, and PMI during treatment were more pronounced in the CRT group than in the NAC group, despite no significant difference in energy and protein intake between the two groups. Although the CRT group included more patients with stage IV disease, similar findings were observed even after excluding these patients. Thus, cancer cachexia alone is unlikely to explain the difference. One possible explanation is that CRT imposes additional physiological stress due to radiotherapy, thereby increasing metabolic demands and nutritional requirements. Although the ESPEN guidelines provide generalized recommendations across patients with cancer, our findings suggest that nutritional needs may vary depending on treatment modality and individual patient condition.

These findings underscore the importance of individual nutritional assessment and management. Close monitoring of serum albumin levels, BMI, and PMI during treatment may help guide timely nutritional interventions and enable tailoring of energy and protein intake to each patient's needs. Incorporating such an individualized approach could potentially improve treatment tolerance and clinical outcomes in patients with ESCC.

The present study had several limitations. First, it was a retrospective study conducted at a single institution in Japan, with a relatively small sample size. Second, we selected healthy controls matched to patients with ESCC by age and sex but did not include additional covariates in the matching process. As a result, there were imbalances between patients with ESCC and healthy controls in terms of dyslipidemia, smoking history, and alcohol consumption. Third, energy and protein intake were estimated based on electronic medical records, which may not fully capture actual intake, especially during outpatient periods. Finally, although we performed subgroup analysis by disease stage, there was variability in baseline characteristics between the CRT and NAC groups, which may introduce residual confounding. Despite these limitations, our findings support the current ESPEN recommendations and emphasize the need for individualized nutritional management based on the treatment modality in patients with ESCC. Future prospective studies are warranted to further clarify optimal nutritional strategies for specific therapeutic contexts.

In conclusion, patients with ESCC had poorer nutritional status than healthy controls, and the nutritional parameters further declined during treatment. Adequate nutritional intake may mitigate this deterioration. Moreover, energy and protein requirements may differ depending on the treatment modality, such as CRT and NAC. These findings highlight the clinical importance of individualized nutritional management to support treatment outcomes in patients with ESCC.

## Author Contributions

**Yumiko Tamai:** data curation (lead), investigation (lead), writing – original draft (equal). **Atsushi Yamada:** methodology (equal), validation (equal), writing – original draft (equal). **Chikatoshi Katada:** methodology (supporting), writing – review and editing (lead). **Masashi Tamaoki:** resources (equal), writing – review and editing (equal). **Motoo Nomura:** resources (equal), writing – review and editing (equal). **Akira Yokoyama:** resources (equal), writing – review and editing (equal). **Hiroyuki Inoo:** resources (equal), writing – review and editing (equal). **Kota Fujii:** resources (equal), writing – review and editing (equal). **Katsuyuki Sakanaka:** resources (equal), writing – review and editing (equal). **Shigeru Tsunoda:** resources (equal), writing – review and editing (equal). **Shinya Ohashi:** resources (equal), writing – review and editing (equal). **Yoshitaka Nishikawa:** methodology (supporting), writing – review and editing (equal). **Mayumi Inoue:** resources (equal), writing – review and editing (equal). **Kazutaka Obama:** resources (equal), supervision (equal), writing – review and editing (equal). **Takashi Mizowaki:** resources (equal), supervision (equal), writing – review and editing (equal). **Manabu Muto:** conceptualization (equal), supervision (lead), writing – review and editing (lead).

## Funding

This research received no specific grants from any funding agency in the public, commercial, or not‐for‐profit sectors.

## Ethics Statement

This study was approved by the Ethics Committee of Kyoto University Graduate School and Faculty of Medicine (R3530‐1).

## Conflicts of Interest

The authors declare no conflicts of interest.

## Supporting information


**FIGURE S1:** Changes in serum albumin levels, BMI, and PMI during CRT and NAC, analyzed separately in each group. Statistical analyses were performed using the Wilcoxon signed‐rank test. BMI, body mass index; CRT, chemoradiotherapy; ESCC, esophageal squamous cell carcinoma; NAC, neoadjuvant chemotherapy; PMI, psoas muscle mass index.

**FIGURE S2:** Correlations between energy and protein intake per kg of body weight during treatment and the post/pre ratio of serum albumin levels, BMI, and PMI in the CRT and NAC groups. Correlations were assessed using Spearman's rank correlation coefficient (rho). BMI, body mass index; CRT, chemoradiotherapy; ESCC, esophageal squamous cell carcinoma; ESPEN, European Society for Clinical Nutrition and Metabolism; NAC, neoadjuvant chemotherapy; PMI, psoas muscle mass index.

**FIGURE S3:** Comparison of post/pre ratios of serum albumin levels, BMI, and PMI between patients with ESCC with insufficient and sufficient energy and protein intake, according to the ESPEN guidelines, analyzed separately for the CRT and NAC groups. Statistical analyses were performed using the Mann–Whitney *U* test. BMI, body mass index; CRT, chemoradiotherapy; ESCC, esophageal squamous cell carcinoma; ESPEN, European Society for Clinical Nutrition and Metabolism; NAC, neoadjuvant chemotherapy; PMI, psoas muscle mass index.

## Data Availability

The data that support the findings of this study are available from the corresponding author upon reasonable request.
