# Supplementary figures and images for: Clinical Implications of Nutritional Intake in Patients With Esophageal Squamous Cell Carcinoma Receiving Chemoradiotherapy and Neoadjuvant Chemotherapy
Source: Cancer Med. 2026 Mar 12;15(3):e71714. doi: 10.1002/cam4.71714 (PMC13093406; doi:10.1002/cam4.71714)

Supplementary figure 1

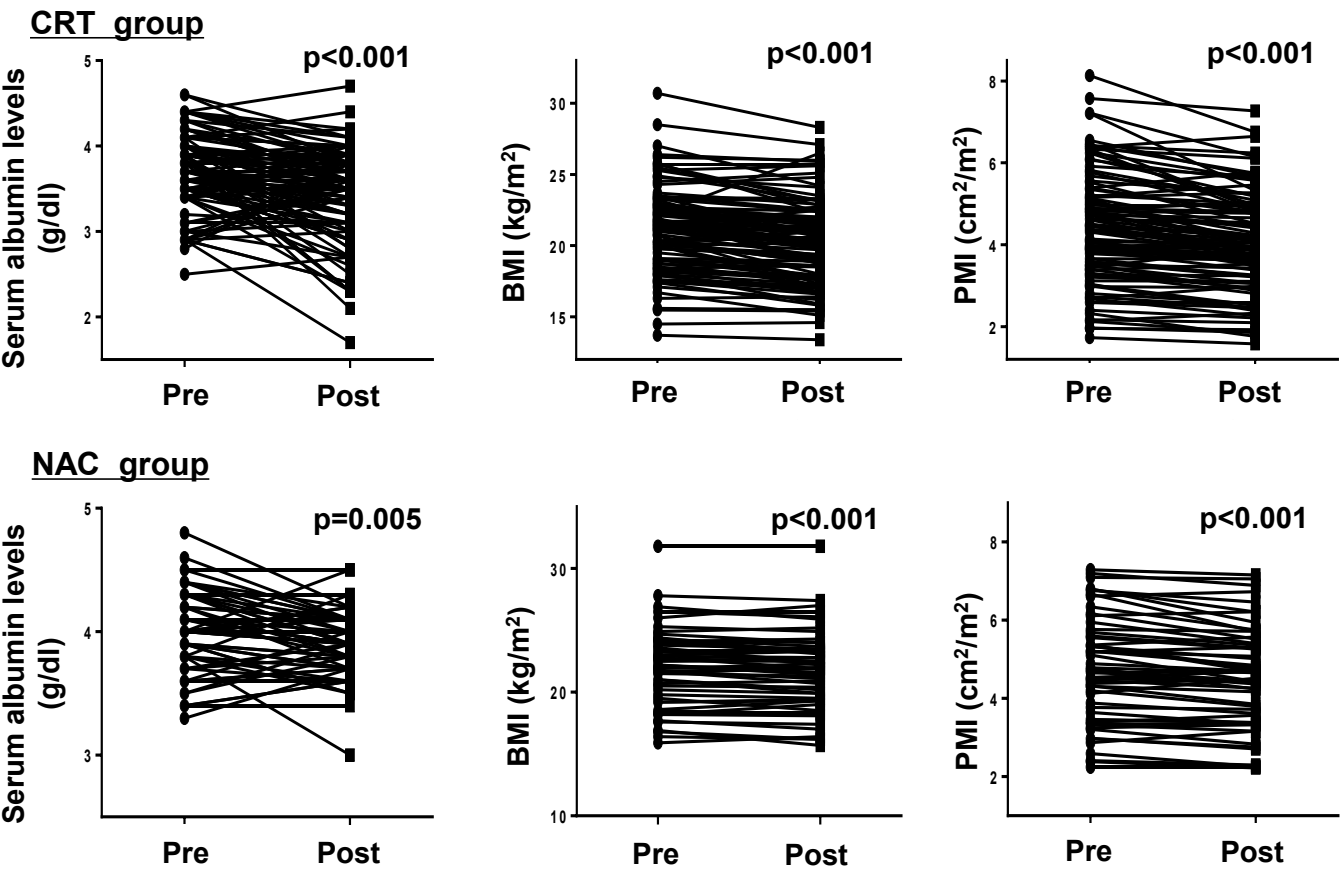

Supplement: Supplementary file 1 — FIGURE S1: Changes in serum albumin levels, BMI, and PMI during CRT and NAC, analyzed separately in each group. Statistical analyses were performed using the Wilcoxon signed‐rank test. BMI, body mass index; CRT, chemoradiotherapy; ESCC, esophageal squamous cell carcinoma; NAC, neoadjuvant chemotherapy; PMI, psoas muscle mass index. [file CAM4-15-e71714-s002.pdf]
