# Supplementary material for: Clinical Implications of Nutritional Intake in Patients With Esophageal Squamous Cell Carcinoma Receiving Chemoradiotherapy and Neoadjuvant Chemotherapy
Source: Cancer Med. 2026 Mar 12;15(3):e71714. doi: 10.1002/cam4.71714 (PMC13093406; doi:10.1002/cam4.71714)

Supplementary figure 2

CRT group

Energy intake

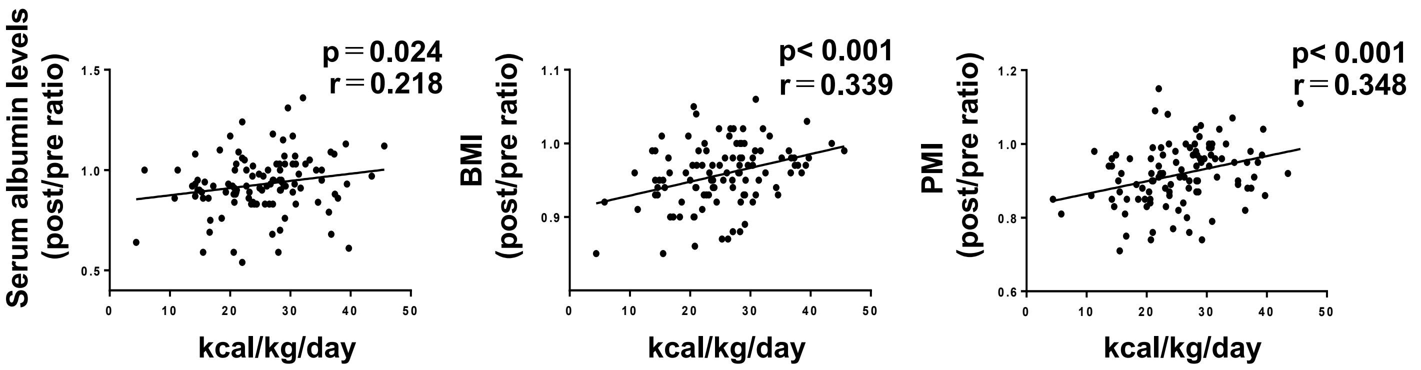

Protein intake

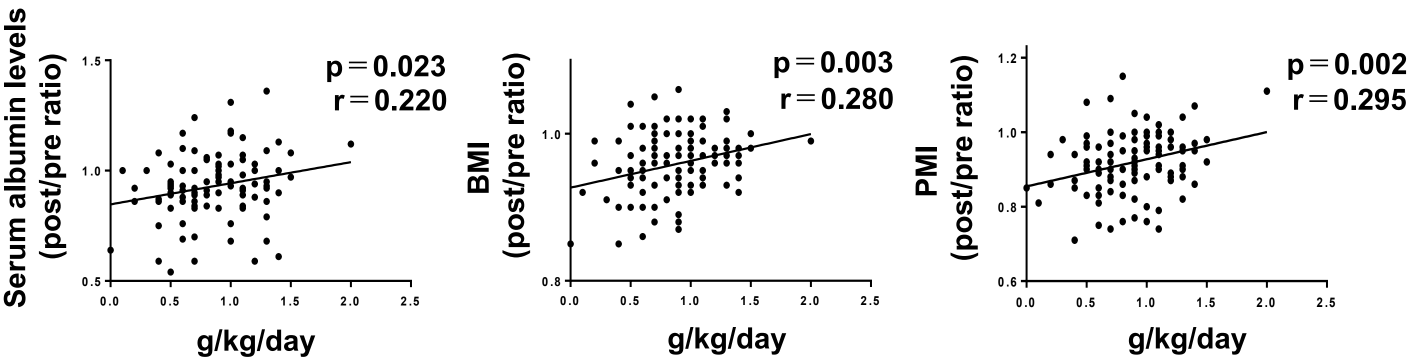

NAC group

Energy intake

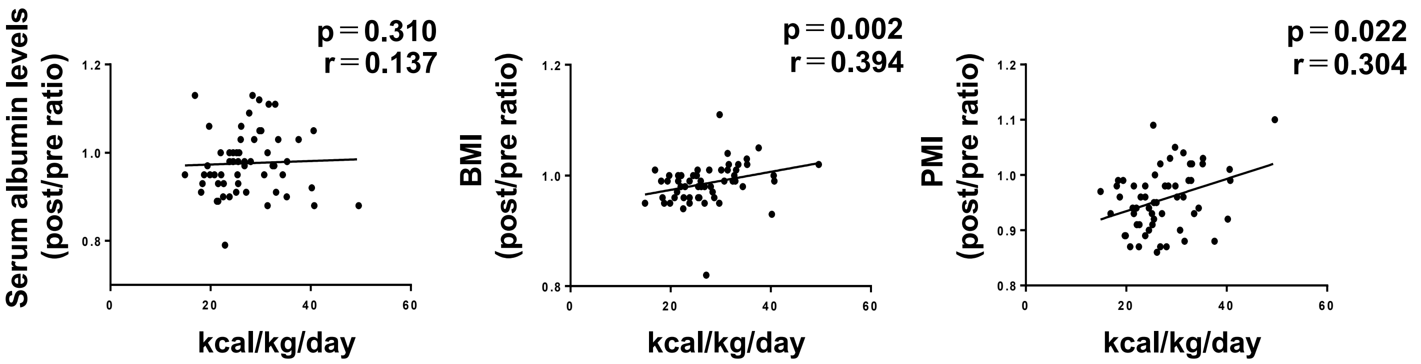

Protein intake

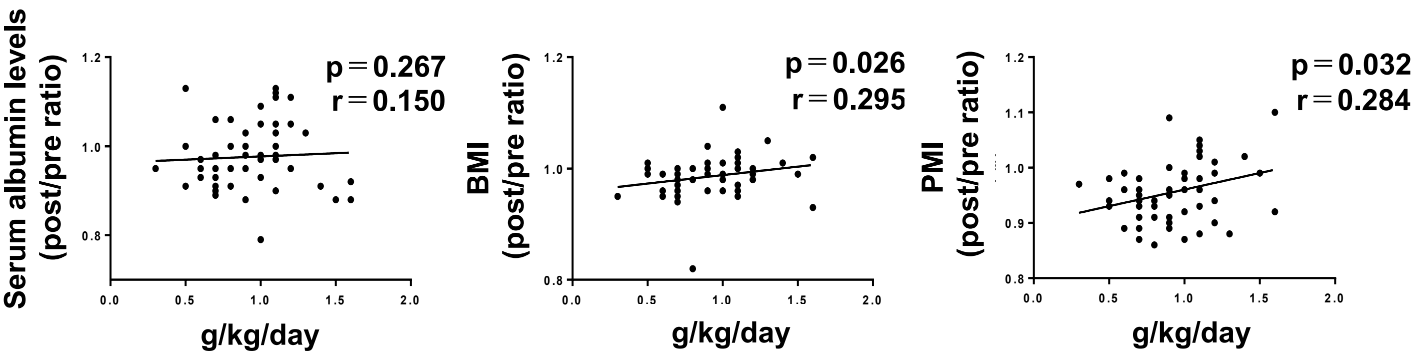

Supplement: Supplementary file 2 — FIGURE S2: Correlations between energy and protein intake per kg of body weight during treatment and the post/pre ratio of serum albumin levels, BMI, and PMI in the CRT and NAC groups. Correlations were assessed using Spearman's rank correlation coefficient (rho). BMI, body mass index; CRT, chemoradiotherapy; ESCC, esophageal squamous cell carcinoma; ESPEN, European Society for Clinical Nutrition and Metabolism; NAC, neoadjuvant chemotherapy; PMI, psoas muscle mass index. [file CAM4-15-e71714-s001.pdf]
