# Supplementary material for: Clinical Implications of Nutritional Intake in Patients With Esophageal Squamous Cell Carcinoma Receiving Chemoradiotherapy and Neoadjuvant Chemotherapy
Source: Cancer Med. 2026 Mar 12;15(3):e71714. doi: 10.1002/cam4.71714 (PMC13093406; doi:10.1002/cam4.71714)

Supplementary figure 3

CRT group

Energy intake

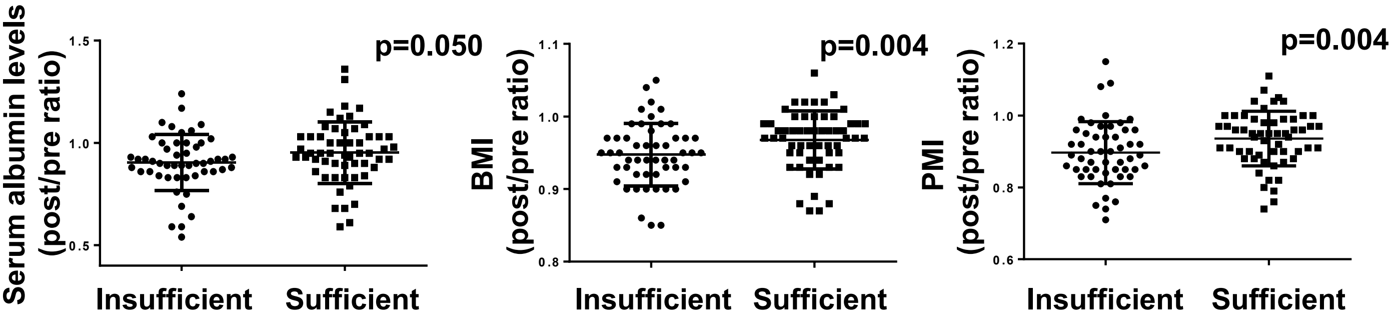

Protein intake

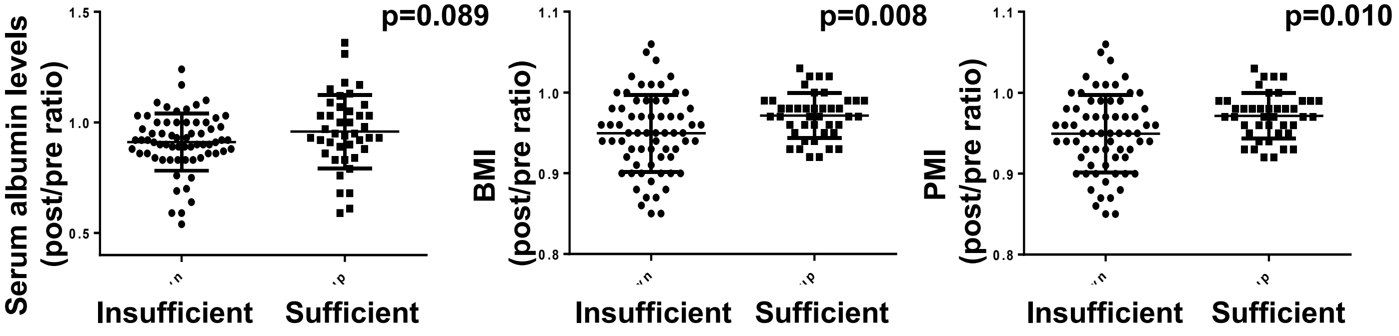

NAC group

Energy intake

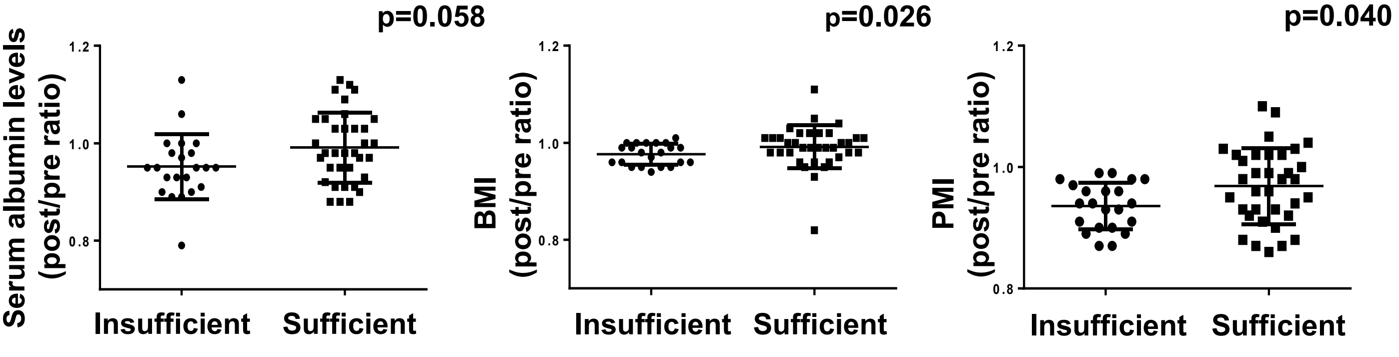

Protein intake

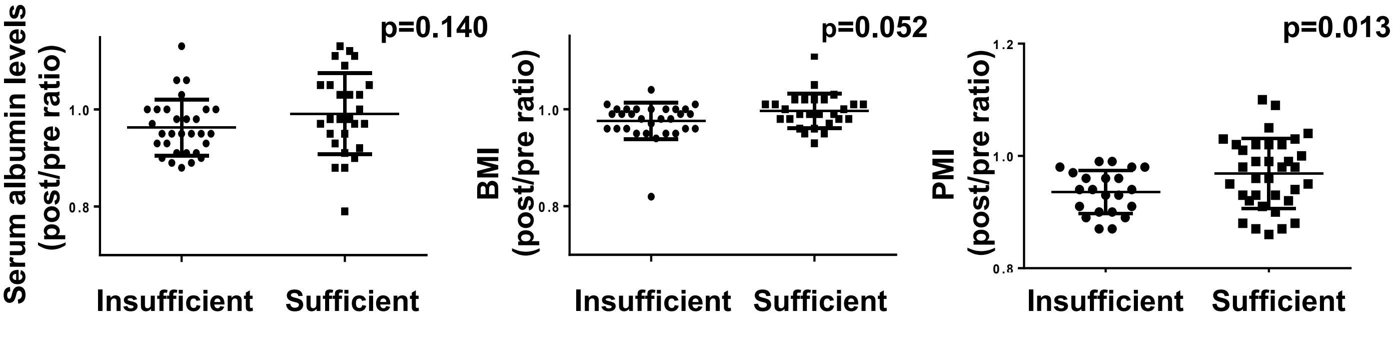

Supplement: Supplementary file 3 — FIGURE S3: Comparison of post/pre ratios of serum albumin levels, BMI, and PMI between patients with ESCC with insufficient and sufficient energy and protein intake, according to the ESPEN guidelines, analyzed separately for the CRT and NAC groups. Statistical analyses were performed using the Mann–Whitney U test. BMI, body mass index; CRT, chemoradiotherapy; ESCC, esophageal squamous cell carcinoma; ESPEN, European Society for Clinical Nutrition and Metabolism; NAC, neoadjuvant chemotherapy; PMI, psoas muscle mass index. [file CAM4-15-e71714-s003.pdf]
